# Optimizing community screening for tuberculosis: Spatial analysis of localized case finding from door-to-door screening for TB in an urban district of Ho Chi Minh City, Viet Nam

**DOI:** 10.1371/journal.pone.0209290

**Published:** 2018-12-18

**Authors:** Luan Nguyen Quang Vo, Thanh Nguyen Vu, Hoa Trung Nguyen, Tung Thanh Truong, Canh Minh Khuu, Phuong Quoc Pham, Lan Huu Nguyen, Giang Truong Le, Jacob Creswell

**Affiliations:** 1 Friends for International TB Relief, Ho Chi Minh City, Viet Nam; 2 Ho Chi Minh City Public Health Association, Ho Chi Minh City, Viet Nam; 3 Go Vap District Preventive Health Center, Ho Chi Minh City, Viet Nam; 4 Ho Chi Minh City Department of Science & Technology, Center for Applied Geographic Information Systems (HCMGIS), Ho Chi Minh City, Viet Nam; 5 Pham Ngoc Thach Hospital, Ho Chi Minh City, Viet Nam; 6 Stop TB Partnership, Geneva, Switzerland; Management Sciences for Health, ETHIOPIA

## Abstract

**Background:**

Tuberculosis (TB) is the deadliest infectious disease globally. Current case finding approaches may miss many people with TB or detect them too late.

**Data and methods:**

This study was a retrospective, spatial analysis of routine TB surveillance and cadastral data in Go Vap district, Ho Chi Minh City. We geocoded TB notifications from 2011 to 2015 and calculated theoretical yields of simulated door-to-door screening in three concentric catchment areas (50m, 100m, 200m) and three notification window scenarios (one, two and four quarters) for each index case. We calculated average yields, compared them to published reference values and fit a GEE (Generalized Estimating Equation) linear regression model onto the data.

**Results:**

The sample included 3,046 TB patients. Adjusted theoretical yields in 50m, 100m and 200m catchment areas were 0.32% (95%CI: 0.27,0.37), 0.21% (95%CI: 0.14,0.29) and 0.17% (95%CI: 0.09,0.25), respectively, in the baseline notification window scenario. Theoretical yields in the 50m-catchment area for all notification window scenarios were significantly higher than a reference yield from literature. Yield was positively associated with treatment failure index cases (beta = 0.12, p = 0.001) and short-term inter-province migrants (beta = 0.06, p = 0.022), while greater distance to the DTU (beta = -0.02, p<0.001) was associated with lower yield.

**Conclusions:**

This study is an example of inter-departmental collaboration and application of repurposed cadastral data to progress towards the end TB objectives. The results from Go Vap showed that the use of spatial analysis may be able to identify areas where targeted active case finding in Vietnam can help improve TB case detection.

## Introduction

Tuberculosis (TB) is one of the most intractable public health challenges and a leading cause of avoidable deaths worldwide. In 2016, there were an estimated 10.4 million incident cases of TB worldwide and 1.7 million TB deaths.[1] Despite advances in treatment and prevention programs, TB incidence is declining only at 1.65% per annum.[2] At this rate, global TB elimination may only be achieved near the end of the 22^nd^ century.[3] A major cause for the slow decline is the estimated 4 million people who develop TB annually who are missed by national TB control programs (NTPs). For this reason, early detection and improved diagnosis of TB is a vital component of WHO’s End TB strategy.[4]

Community-based screening for TB can detect more people with TB than passive case finding alone and detect them earlier.[5] However, strategies to improve case detection and their results will differ by setting.[6] As a result, the population-level impact of these activities remains poorly understood.[7] For example, screening of household contacts and people living with HIV is efficient and recommended in all settings[5], but due to the limited size of these high-risk populations the impact on additional cases identified, i.e., cases detected over baseline[8], is limited. Contacts and PLHIV also have a higher likelihood of detection through routine case finding activities.[9,10] Active screening in other high-risk groups such as mine workers, prisons, refugees and diabetics has also been recommended, but these strategies suffer from similar limitations in coverage and lower yields than contacts and PLHIV.[11–15] Facility-based screening can produce a high yield, but depends on patient-initiated health-seeking usually in advanced stages of disease progression and thereby may have limited impact on transmission.[16–19] Conversely, indiscriminate population-wide screening and mass chest x-ray screening produce broad coverage, but have traditionally produced a low yield of newly diagnoses tuberculosis.[20] Driven by a confluence of complexities and risk heterogeneities, and the associated high marginal cost of detection[21,22], WHO has recommended to avoid the pursuit of resource-intensive, unfocussed strategies, particularly involving mass radiography.[23] Identifying “middle-ground” solutions between high yield activities such as contact investigation and high coverage ones including community screening or active case finding (ACF) often mark successful approaches.[24–26]

Considerable evidence has shown that proximity and prolonged exposure to a source case, for example in households, among community contacts, places of mass congregation or other disease clusters, greatly raises the risk of TB infection and progression to active disease.[27–34] With the advance of Global Positioning Systems (GPS), precision vector cartography and mobile communication technology, there is a growing body of evidence on the spatial heterogeneity of tuberculosis prevalence to establish the value of Geographic Information Systems (GIS) in TB surveillance and to elucidate transmission dynamics in such disease “hotspots.”[35–39] Many studies and approaches further incorporate molecular genetic, demographic,[40] socioeconomic or other subnational data to contextualize these disease clusters.[41,42] However, there remains limited quantitative evidence on the potential localized TB burden in geographic proximity to index patients, possible cluster size of such TB hotspots or the number needed to screen to find a TB case within these disease clusters.[43,44] We similarly lack evidence on targeting proximal neighbor(hood) contacts for TB screening.[5,45,46] This opens up the avenue to explore “catchment areas” as means to quantify the number needed to screen (NNS) and theoretical yield from targeted ACF. However, to apply spatial restrictions methods such as catchment areas using the concentric circle approach will require the ability to quantify the potential effectiveness and to assess the associated resource implications.[47–49]

The advent of GIS and mobile communications technology in low- and middle-income countries warrants applying them to screening activities. We conducted an exploratory analysis of spatial and temporal relations of notified TB cases in an urban district of Ho Chi Minh City, Viet Nam, to quantify the search parameters, i.e., size of the area and incubation time after notification, and propose a pragmatic neighborhood contact screening strategy that could improve coverage while maintaining an acceptably high yield.

## Methods

### Study design & aims

This study was a retrospective cross-sectional, spatial analysis of routine TB surveillance and digital cadastral data. The aim of this study was to determine the existence and size of a catchment area around an index case, in which door-to-door screening could theoretically yield significantly more cases compared to population-wide screening. Our objectives were to assign individual GPS coordinates to all index cases in our sample and calculate the average theoretical yield from simulated door-to-door screening in three catchment area sizes, which we then compared to a published reference value. Lastly, we identified secondary index case parameters that were positively and negatively associated with theoretical yield.

### Study setting

The study took place in Go Vap district, Ho Chi Minh City (HCMC). Go Vap is an urban district with a population of 650,000 people in an area of ~21km^2^ for a population density of approximately 31,000 persons per km^2^. As such, Go Vap is one of the most densely population areas in HCMC (4,020 persons/km^2^) and Viet Nam (290 persons/km^2^) overall. In 2014, 768 TB cases were notified in Go Vap for a notification rate of 136.7 per 100,000 people compared to 188.3 in HCMC and 112.8 in Vietnam.[50]

### Data sources & processing

The Center for Applied GIS of Ho Chi Minh City within the HCMC Department of Science and Technology has a GIS database of the city with detailed geospatial vector data (shapefiles) of real estate property lots.[51] In this study, we repurposed this database, which typically informs urban planning and construction projects, for public health use. We included all drug-sensitive and drug-resistant TB cases notified in Go Vap between 1 November 2011 and 30 November 2015 with a recorded address in the district. These patients served as index cases for analysis throughout the study. We excluded patients re-enrolled immediately, i.e., within 1 month subsequent to a recorded treatment failure. We automated geocoding of patient addresses using a matching algorithm, manually geocoded addresses that did not produce an exact match. We excluded patients from the sample for whom automated and manual geocoding was unsuccessful. Using the GIS software, we then calculated concentric catchment areas around each patient’s residence with radii of 50m, 100m and 200m. We selected these discrete radii as they corresponded to the amount of time (a week, a month and a quarter, respectively) an outreach worker needed to screen all households based on prior experience.[52] A catchment area with radius 50m included on average 74.5 (IQR: 49–95) households, while areas of 100m and 200m included an average of 250.5 (IQR: 184–311) and 873.9 (IQR: 696–1062) households, respectively.

For each index case, we counted the number of TB cases who resided inside the three catchment areas and who were notified after the index case within three predefined notification windows (one, two and four quarters). We used the total number of real estate property lots as a proxy for households in each catchment area. We counted a property lot to lie within the catchment area, if it included any part of the property boundary vectors. We multiplied the national average urban household size with the number of property lots to enumerate the estimated population in each catchment area.[53] We calculated the theoretical yield from door-to-door screening in these areas as the proportion of notified TB cases over the estimated number of residents in each catchment area (S1–S4 Figs).

### Data analysis

We calculated summary statistics for the counts of notifications and households, and descriptive statistics for the patient covariates in the sample. The dataset consisted of multidimensional panel data with nine repeated measures for each index case (three catchment areas and three notification windows). The response variable, theoretical yield for each index case and catchment area-notification window combination, showed a semi-continuous, negative binomial distribution. We used generalized estimating equation (GEE) methods to adjust standard errors for non-normality and within-subject correlation of the repeated measures.[54,55] We chose GEE methods over mixed effects models due to the time-invariant nature of the study and its parameters, the large sample with limited repeated measures and the population-level nature of the response variable.[56] Given its continuous nature and the lack of missing data in the primary exposure and response variables, the analyses were not exposed to typical GEE limitations.[57] We used univariate regression to describe the association of theoretical yield (primary response) and catchment area size and notification window (primary exposures), and secondary covariates. Aside from age and gender, secondary covariates with a p-value of less than 0.2 in the univariate GEE regression model were fitted in the multivariate model. The model accounted for interaction between the two primary exposure variables. Based on the Quasi-likelihood Information Criterion (QIC) we used an exchangeable correlation structure and a Gaussian variance function as model specifications and calculated localized, theoretical detection yields from the model’s coefficients.[58,59] We expressed these theoretical TB detection yields in terms of number needed to screen (NNS), which was calculated as the inverse of the theoretical yield.

### Ethical considerations

We obtained written permission for analysis of the TB patient data from the Go Vap District Preventive Health Center, the administrative authority of the District TB Unit and legal owner of the data. The ethics committee of the Ho Chi Minh City Provincial HIV/AIDS Committee provided ethical approval for this study.

## Results

In the period from 2011 to 2015, the Go Vap District TB Unit notified 3,133 TB people with TB. Among these, 79 people were retreatment cases who enrolled immediately after treatment failure and did not meet the inclusion criteria. We geocoded 2,513 (82%) cases automatically and 533 (18%) manually locating them at the nearest main street and primary alley. We were unable to geocode 8 addresses, so the final sample size included 3,046 people (99% of those notified).

Table 1 shows descriptive statistics of the sample. About one-third (n = 976) of notified cases were female and median age was 40 years (IQR: 28–52) years. People with TB/HIV coinfection comprised 6% (198) of cases, while 5% (145) reported comorbid diabetes. The majority of the sample was unemployed (42%, 1,275) or employed as unskilled or semi-skilled labor (44%, 1,320). Temporary residents comprised 29% (869) of the sample, among whom the majority (675) consisted of short-term, inter-province migrants, defined as persons whose household registration is in a district or province different from the one in which they currently reside. The median distance to the DTU was 2.8km (IQR: 1.5, 3.5). Smear-positivity characterized 54% (1,631) of cases, among whom 2% (39) were diagnosed with MDR-TB while about a quarter of the cases were extra-pulmonary TB. Previously untreated cases comprised 78% (2,377) of the sample. Treatment outcomes were high with 85% (2,607) having documented treatment success.

**Table 1:** Patient characteristics of notified TB cases at the Go Vap district TB Unit, Ho Chi Minh city, Viet Nam (n = 3,046^¥^).

	Total N (%)	(cont.)	Total N (%)
Total	3,046 (100)	Total	3,046 (100)
Sex		Type of TB^§^	
Female	976 (32)	AFB(+)	1,631 (54)
Male	2,057 (68)	AFB(−)	679 (22)
Age		EP	736 (24)
<25 years	500 (16)	Drug-resistant	
25–34 years	707 (23)	No	3,007 (99)
35–44 years	582 (19)	Yes	39 (1)
45–54 years	599 (20)	Patient type	
≥55 years	645 (21)	New	2,377 (78)
HIV/AIDS ^¶^		Relapse	304 (10)
No/Unknown	2,848 (94)	Failure	39 (1)
Yes	198 (6)	LTFU retreatment#	32 (1)
Diabetes mellitus		Transfer In┼	294 (10)
No/Unknown	2,901 (95)	Treatment outcomes	
Yes	145 (5)	Success	2,607 (85)
Employment		Cure	1,344 (44)
Unemployed	1,275 (42)	Complete	1,263 (41)
Un-/semi-skilled	1,320 (44)	LTFU#	121 (4)
Skilled	411 (14)	Failure	99 (3)
Residency		Death	114 (4)
Permanent	2,177 (71)	Transfer out	102 (3)
Long-term intra-province	184 (6)	Proximity to TB Unit	
Long-term inter-province	10 (0)	Close	1,002 (34)
Short-term inter-province	675 (22)	Medial	1,028 (35)
		Distant	927 (31)

Notes

¥ Individual parameters may include missing data, which were excluded from the regression analysis

¶ Human Immunodeficiency Virus/Acquired Immunodeficiency Syndrome

§ AFB(+) = Sputum smear positive; AFB(−) = Sputum smear negative; EP = Extra-pulmonary TB

# LTFU = Loss to Follow-up

┼ Inbound transfers and referrals with prior uncertain exposure to anti-TB drugs.

Based on the fitted model, the adjusted theoretical yield of localized door-to-door screening in the base scenario with a notification window of 2 quarters was 0.32% (95%CI: 0.27, 0.37) in a catchment area with radius 50m. For catchment areas with radii of 100m and 200m, the theoretical yields were 0.21% (95%CI: 0.14, 0.29) and 0.17% (95%CI: 0.09, 0.25), respectively (Table 2). This corresponds to NNS of 313 (95%CI: 270–370), 476 (95%CI: 345–714) and 588 (95%CI: 400–1,111), respectively.

**Table 2:** Adjusted theoretical yield by catchment area size and notification window based on the GEE linear regression model (n = 3,046).

	Radius = 50m	Radius = 100m	Radius = 200m
*Theoretical yield┼* *in all catchment areas (mean %*, *95%CI)*			
1 Quarter	0.23 (0.17, 0.30)	0.12 (0.04, 0.21)	0.08 (0.00, 0.17)
2 Quarters¶	0.32 (0.27, 0.37)	0.21 (0.14, 0.29)	0.17 (0.09, 0.25)
4 Quarters	0.47 (0.40, 0.53)	0.36 (0.27, 0.45)	0.32 (0.22, 0.41)
*Number needed to screen┼* *in all catchment areas (mean NNS*, *95%CI)*			
1 Quarter	435 (333, 588)	833 (476, 2500)	1250 (-, 10000)
2 Quarters¶	313 (270, 370)	476 (345, 714)	588 (400, 1111)
4 Quarters	213 (189, 250)	278 (222, 370)	313 (244, 455)
*Zero-yield catchment areas (proportion %*, *95%CI)*			
1 Quarter	78.6 (77.1, 80.0)	55.9 (54.1, 57.6)	22.1 (20.6, 23.6)
2 Quarters¶	65.7 (64.0, 67.4)	37.6 (35.9, 39.3)	10.6 (9.6, 11.8)
4 Quarters	53.0 (51.1, 54.7)	24.2 (22.7, 25.8)	6.0 (5.2, 6.9)
*Theoretical yield┼* *in non-zero catchment areas§* *(mean %*, *95% CI)*			
1 Quarter	0.69 (0.60, 0.77)	0.20 (0.05, 0.35)	0.09 (0.00, 0.25)
2 Quarters¶	0.77 (0.70, 0.85)	0.28 (0.15, 0.42)	0.18 (0.03, 0.32)
4 Quarters	0.93 (0.84, 1.02)	0.44 (0.29, 0.59)	0.33 (0.17, 0.49)
*Number needed to screen┼* *in non-zero catchment areas§* *(mean NNS*, *95% CI)*			
1 Quarter	145 (130, 167)	500 (286, 2000)	1111 (-, 1428)
2 Quarters¶	130 (118, 143)	357 (238, 667)	556 (312, 3333)
4 Quarters	108 (98, 119)	227 (169, 345)	303 (204, 588)

Notes

┼ Based on the national average urban household size of 3.66 persons per household; stratified by notification windows of 1, 2 and 4 quarters subsequent to the index case

¶ Base case scenario

§ Refers to catchment areas that have at least one notification in addition to the index cases in any of the three radii and notification windows

The majority of scenarios had a high proportion of zero-yield catchment areas, i.e., contained no other notification in the catchment area aside from the index case. The proportion of zero-yield catchment areas ranged from 53.0%-78.6% for catchment areas of radius 50m and declined to ranges of 24.2%-55.9% and 6.0%-22.1% for radii of 100m and 200m, respectively. The theoretical yield in non-zero yield catchment areas increased to 0.77% (95%CI: 0.70%-0.85%), 0.28% (95%CI: 0.15%-0.42%), and 0.18% (95%CI: 0.03%-0.32%) for catchment areas of 50m, 100m and 200m, respectively. This corresponds to NNS of 130 (95%CI: 118–143), 352 (95%CI: 237–683) and 571 (95%CI: 313–3,225), respectively.

In addition to the strong association between theoretical yield and the two primary exposures, catchment area size and notification window, results from the fitted GEE linear regression model in Table 3 displayed associations between theoretical yield and other index patient characteristics. The model showed a significant negative association between theoretical yield and distance to DTU (beta = -0.02, p<0.001) higher theoretical yield among short-term inter-province migrants (beta = 0.06, p = 0.022) and among people for whom treatment failed (beta = 0.12, p = 0.001).

**Table 3:** GEE linear regression model of theoretical yield of door-to-door screening adjusted for primary and secondary index patient characteristics (n = 3,046).

	Coefficient	95% CI	p-value╠
Constant	0.32**	0.27,0.37	<0.001
Catchment area (CA)			
100m	-0.11**	-0.13,-0.08	<0.001
200m	-0.15**	-0.18,-0.12	<0.001
Notification window (NW)			
1 quarter	-0.09**	-0.10, -0.08	<0.001
4 quarter	0.15**	0.14, 0.16	<0.001
Female	0.02	-0.02, 0.06	0.340
Age	0.00	-0.00, 0.00	0.462
Patient type			
Relapse	0.01	-0.03, 0.05	0.683
Retreatment after failure	-0.05	-0.10, 0.00	0.059
LTFU retreatment#	0.09	-0.13, 0.31	0.428
Inbound transfer┼	0.04	-0.09, 0.18	0.530
Unknown prior treatment	-0.03	-0.06, 0.09	0.140
Residency status			
Long-term intra-province	0.01	-0.05, 0.06	0.832
Long-term inter-province	-0.05	-0.15, 0.05	0.328
Short-term inter-province	0.06**	0.01, 0.11	0.022
Treatment outcome			
Treatment success	-0.01	-0.04, 0.02	0.515
Failure	0.12**	0.05, 0.18	0.001
Death	0.00	-0.04, 0.04	0.897
Loss to follow-up	0.13	-0.05, 0.31	0.148
Transfer out	-0.03	-0.09, 0.02	0.205
Distance to DTU¥	-0.02**	-0.03, -0.01	<0.001

Notes

** Reject the null hypothesis at a 95% confidence level

╠ Wald test

# LTFU = Loss to Follow-up

┼ Inbound transfers and referrals with prior uncertain exposure to anti-TB drugs

¥ Continuous variable with distance in kilometers.

## Discussion

This was an exploratory study using the combination of routine TB surveillance and urban planning data to explore opportunities to optimize active case finding for TB. We found no other studies that have attempted this enumeration at the level of index patient household or individual catchment area.

It is clear that ACF will be needed to reach the people with TB that are currently missed by facility-based case finding NTPs use since their reach is limited.[1,4,7,9,60] However, ACF is inherently more expensive and indiscriminate measures are not productive.[61–64] Approaches that improve the yield of ACF interventions are needed. We used estimated localized notification rates, i.e., TB cases notified over the total estimated population in a catchment area, to illustrate spatial heterogeneity and the existence of TB disease clusters in our sample as an alternative to standard spatial autocorrelation methods, which may be included in further analyses.[65,66] In a review of ACF interventions, the weighted mean NNS for community and population-wide screening was 603 corresponding to a detection yield of 0.17%.[67] The theoretical yields in the 50m catchment area size across all notification window scenarios were significantly higher (S5 Fig), but on their own, are unlikely to merit community screening. Our results suggest that hotspots may be identified even in catchment areas with 100m and 200m radii in densely populated urban settings, if a sufficiently long notification window were permitted.

An important consideration for the economic viability of this type of spatially restricted door-to-door screening involves the identification and avoidance of zero-yield catchment areas. Our results showed that neighborhood contact screening in a catchment area of 50m within a quarter of notification would have yielded no additional case in almost four-fifths of index patients. Targeting the correct index cases and avoiding zero-yield catchment areas increased yield in our study 2–3 times. Given the rudimentary nature of the routine surveillance data available for this study, we identified only three index patient covariates that were significantly associated with theoretical yield and may improve targeting of the right catchment areas. One of these parameters was treatment failure of the index patient. This parameter was positively associated with yield, possibly linked to prolonged transmission. Short-term, temporary residency status was also associated with non-zero community cases which may be a function of the propensity of economic migrants to reside in boarding homes and urban slum communities upon arrival in the city.[68–72] Concordant with other studies, temporary residency may be an appropriate indicator for the higher likelihood of finding a TB hotspot.[73–77] The third significantly, albeit negatively, associated parameter was index case distance to the District TB Unit. This finding may relate to our use of routine notifications, which evidence has shown to be lower at greater distances from the TB treatment facility.[78,79] As such, this result may imply a localized under-detection rather than under-representation of TB patients.

In summary, the results of this study suggest that conducting door-to-door screening in a 50m radius around an index case with temporary residency status and history of treatment failure may be an economically viable strategy to expand coverage at acceptable case detection yields. In concordance with our results, studies have similarly evaluated and identified neighborhood contacts [80], and specifically those within 50m of an index case [43], to comprise a viable target population for intensified screening with productive yields.

While the government of Viet Nam passed legislature with the goal of reducing TB prevalence to 20 per 100,000 by 2030, the current prevalence and rate of reduction of 4.6% suggest that the country may miss the projected deadline by over three decades.[81,82] Implementing neighborhood screening in 50m catchment areas around retreatment and migrant index cases may be one rapidly implementable strategy to bend the curve. This strategy may also be applicable outside of Viet Nam in other high-burden countries with similar urbanization trends and sociocultural attributes. Studies have shown that proximal clustering of first-degree relatives and high degrees of social interaction in the immediate neighborhood and neighborhood establishments, e.g., bars, cafes, karaoke shops, are significant contributors to tuberculosis transmission, particularly in high burden settings.[83–86]

However, given the limited geographic scope and retrospective nature of the study, further research on this topic seems warranted. A follow-up study on this subject could aim to validate prospectively the theoretical yields obtained from our analysis. Such a prospective study may employ rapid molecular diagnostics instead of smear microscopy for diagnosis of TB in the neighborhood contact and genotypic fingerprinting for validation of the index case as the source of transmission. The prospective study could further evaluate interventions with different types of intensity. For example, the study could evaluate the effectiveness and cost effectiveness of screening a catchment area using an approach based on mobile radiography units rather than door-to-door screening by community health workers.

An inherent inaccuracy of this study is that theoretical yield should be discounted for cases notified through routine case finding over time. In our sample of routine notification data from 2011–2015 in Go Vap, we identified a total of 356 (12% of total notifications) household contacts living in 170 households. Of the 170 households, 155 (91%) included the index case and one other notified household contact. In 14 (8%) households there were three notified patients and one household contained four notified cases. Identifying households with multiple notified cases may help identify “super-spreaders” for whom more intensified outbreak investigation may be warranted.[87,88]

A nationally representative cluster-randomized controlled trial on facility-based household contact investigation conducted in Viet Nam reported a relative risk between active and routine household contact investigation of 2.5, suggesting that approximately 40% of household contacts may be notified through routine case finding, while the remainder would have been missed or detected later.[9] We re-analyzed the dataset excluding all 356 household contact notifications. The theoretical yields of this subset across all nine catchment area-notification window scenarios did not change significantly. This suggests that household contact investigation may not affect the yield of catchment area screening at a population level, likely due to the limited proportion of TB cases stemming from intra-household transmission compared to other community sources in moderate and high prevalence settings.

This study has several limitations. The theoretical yields are based on passive notification in the public sector, primarily detected with microscopy, all factors associated with under-detection of incident cases[1,89,90], meaning the yields are likely underestimates. One of the variables explaining lack of community cases was distance to the health facility, which may also mean that people with TB were missed by the passive system rather than a true association with fewer community cases since other studies measuring this have shown similar results.[75] In addition, we did not differentiate between residential and commercial property lots. We were also not able to differentiate between property lots with single-family or congregate housing, particularly informal boarding home communities. The analysis did not take into consideration property lot sizes for the population estimate of the catchment areas. These factors may have contributed to an over- or underestimation of the total number of residents in a catchment area and subsequently theoretical yield. However, uncertainties may have been mitigated by the large sample size of index cases and high granularity in the cadastral data. Transmission patterns via genotyping of notified cases and health-seeking behaviors through additional primary data collection as used in similar spatial analysis studies may help our understanding of the results.[91,92]

## Conclusions

To reach the people with TB currently missed by NTPs, we need new strategies that detected people with TB earlier and in greater numbers. There is strong agreement that eliminating TB will require intensified TB case finding beyond the status quo. Using geospatial mapping to create models to enhance theoretical case finding yields may be useful to optimize active case finding approaches.

## Supporting information

S1 Data Dataset. Go Vap index case dataset 2011–2015 with counts and proportions of households and TB notifications by catchment area and notification windows, and secondary index case parameters.
(CSV)

S1 Fig TB notifications in Go Vap district, 2011–2015. Visualization of geocoded index patients with residency in Go Vap district notified at the district TB unit from 2011–2015.
(TIF)

S2 Fig TB notifications in a sub-segment of Go Vap district. Visualization of a select number of geocoded index patients in several wards in Go Vap district.
(TIF)

S3 Fig TB notifications layered onto cadastral property lot data. Visualization of households and incident TB cases within specified catchment areas and notification window (here: r = 100m, t = 1 quarter).
(TIF)

S4 Fig Sample localized household and patient density by the primary study exposure parameters. Cross-tabulation of the total count of property lots as well as the count and proportion of TB notifications around a sample index case. These data are tabulated by catchment area and by time window, specifically the full timeframe, 2011–2015, and the three notification window scenarios.
(TIF)

S5 Fig Theoretical yield comparison with literature. Theoretical yield of door-to-door screening by catchment area and notification window compared to pooled estimates from literature (n = 3,046).
(TIF)
