# Supplementary figures and images for: Optimizing community screening for tuberculosis: Spatial analysis of localized case finding from door-to-door screening for TB in an urban district of Ho Chi Minh City, Viet Nam
Source: PLoS One. 2018 Dec 18;13(12):e0209290. doi: 10.1371/journal.pone.0209290 (PMC6298730; doi:10.1371/journal.pone.0209290)

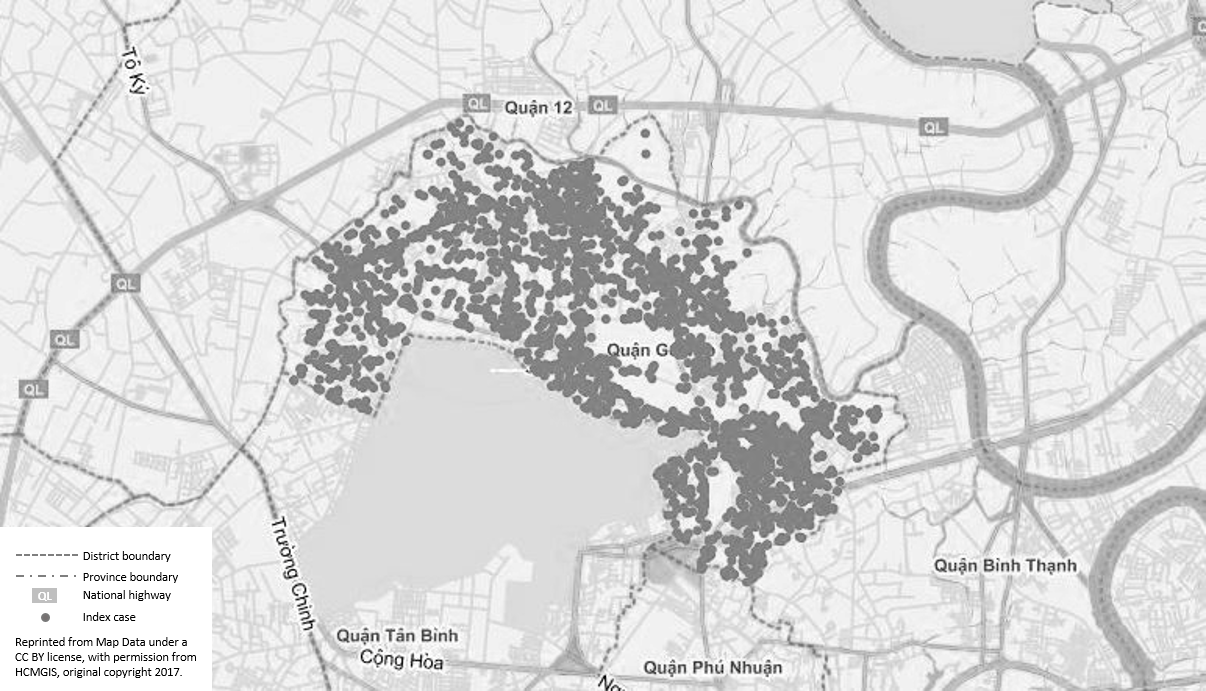

Supplement: S1 Fig — Visualization of geocoded index patients with residency in Go Vap district notified at the district TB unit from 2011–2015. (TIF) [file pone.0209290.s002.tif]

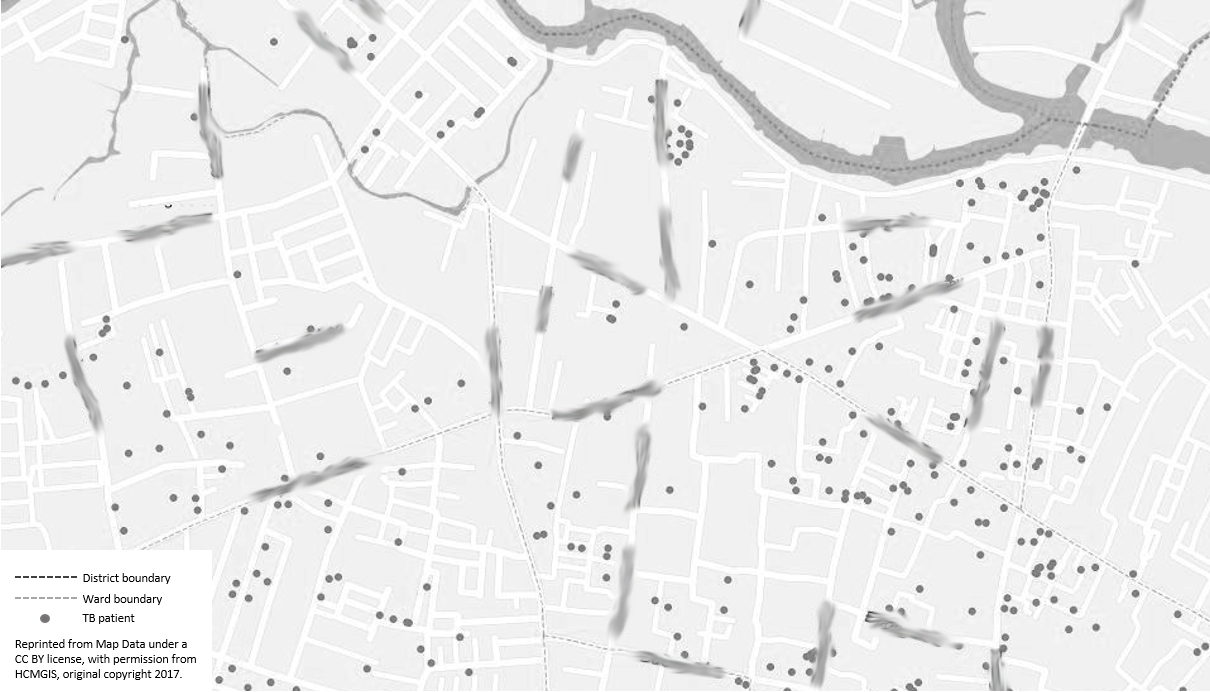

Supplement: S2 Fig — Visualization of a select number of geocoded index patients in several wards in Go Vap district. (TIF) [file pone.0209290.s003.tif]

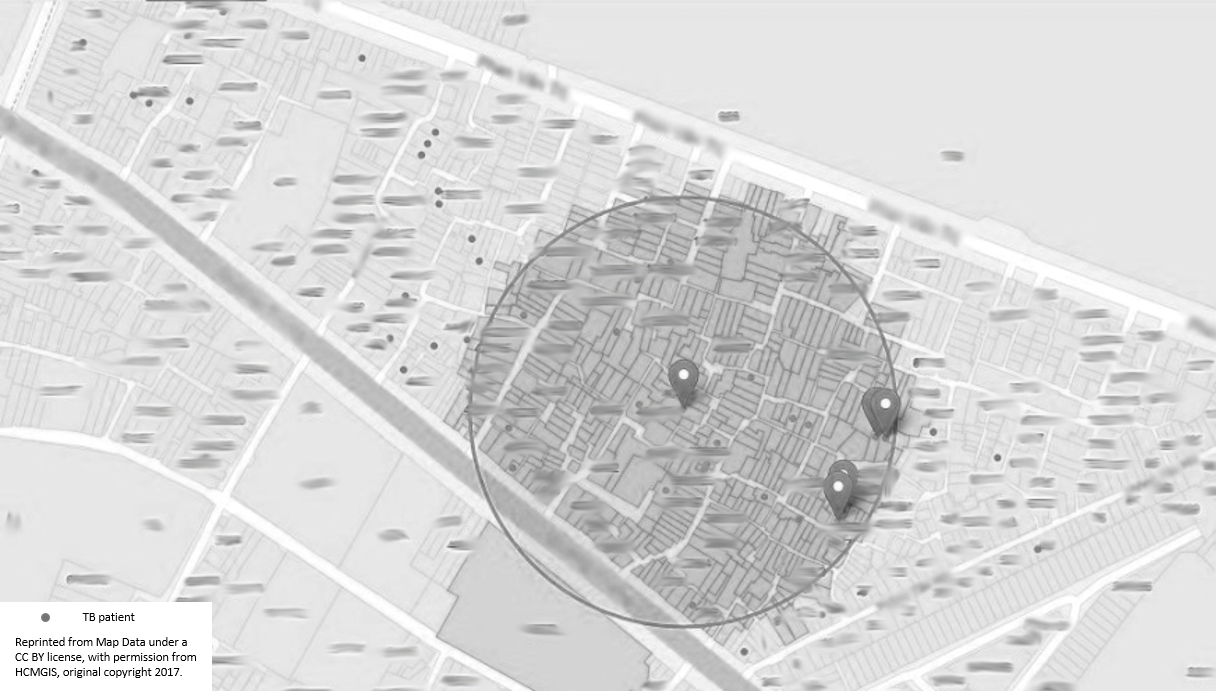

Supplement: S3 Fig — Visualization of households and incident TB cases within specified catchment areas and notification window (here: r = 100m, t = 1 quarter). (TIF) [file pone.0209290.s004.tif]

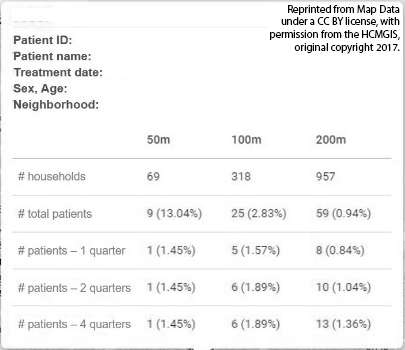

Supplement: S4 Fig — Cross-tabulation of the total count of property lots as well as the count and proportion of TB notifications around a sample index case. These data are tabulated by catchment area and by time window, specifically the full timeframe, 2011–2015, and the three notification window scenarios. (TIF) [file pone.0209290.s005.tif]

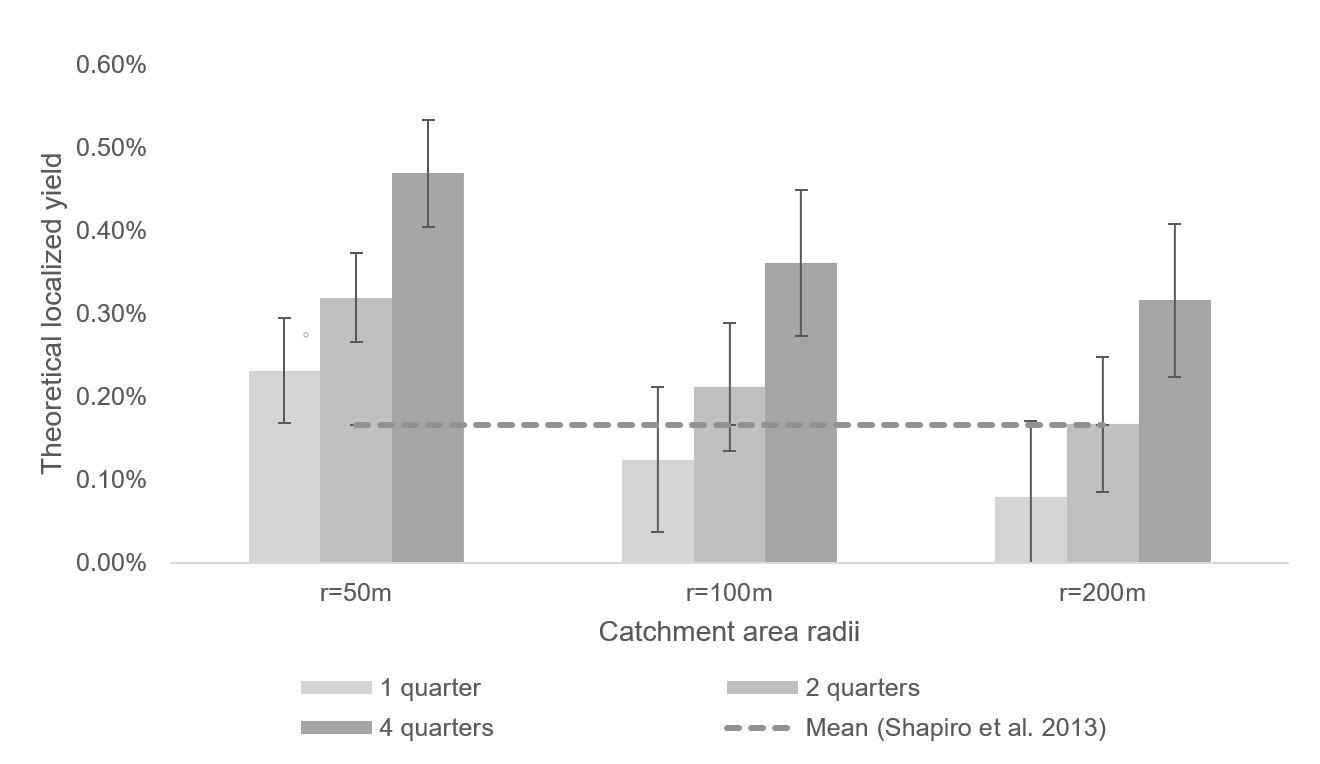

Supplement: S5 Fig — Theoretical yield of door-to-door screening by catchment area and notification window compared to pooled estimates from literature (n = 3,046). (TIF) [file pone.0209290.s006.tif]
